# A biologically active peptide mimetic of N-acetylgalactosamine/galactose

**DOI:** 10.1186/1756-0500-2-23

**Published:** 2009-02-11

**Authors:** Laura L Eggink, J Kenneth Hoober

**Affiliations:** 1 Faculty of Biomedicine and Biotechnology, School of Life Sciences, Arizona State University, Tempe, AZ 85287-4501, USA

## Abstract

**Background:**

Glycosylated proteins and lipids are important regulatory factors whose functions can be altered by addition or removal of sugars to the glycan structure. The glycans are recognized by sugar-binding lectins that serve as receptors on the surface of many cells and facilitate initiation of an intracellular signal that changes the properties of the cells. We identified a peptide that mimics the ligand of an N-acetylgalactosamine (GalNAc)-specific lectin and asked whether the peptide would express specific biological activity.

**Findings:**

A 12-mer phage display library was screened with a GalNAc-specific lectin to identify an amino acid sequence that binds to the lectin. Phage particles that were eluted from the lectin with free GalNAc were considered to have been bound to a GalNAc-binding site. Peptides were synthesized with the selected sequence as a quadravalent structure to facilitate receptor crosslinking. Treatment of human peripheral blood mononuclear cells for 24 h with the peptide stimulated secretion of interleukin-8 (IL-8) but not of IL-1β, IL-6, IL-10, or tumor necrosis factor-α (TNF-α). The secretion of IL-21 was stimulated as strongly with the peptide as with interferon-γ.

**Conclusion:**

The data indicate that the quadravalent peptide has biological activity with a degree of specificity. These effects occurred at concentrations in the nanomolar range, in contrast to free sugars that generally bind to proteins in the micro- to millimolar range.

## Background

Many cells express cell-surface receptors that bind sugar-containing ligands and serve important regulatory functions [1]. Extensive research over the past two decades has been devoted to design of peptide mimetics of sugars [2] to serve as vaccines that elicit anti-carbohydrate antibodies [3,4] or to bind with high affinity to specific antibodies [5,6]. We asked whether a peptide mimetic of N-acetylgalactosamine (GalNAc) could be identified that induces specific responses. For this purpose, a phage display library was screened with a GalNAc-specific lectin as a receptor analog. A consensus amino acid sequence emerged in the variable region of the pIII protein in the selected phage particles. Because clusters of GalNAc bind to receptors with higher affinity than a single residue [7], and receptor crosslinking is often required for many signal transduction mechanisms [8], we designed and tested a multivalent structure containing this sequence.

## Selection and synthesis of a GalNAc mimetic

The lectin from *Helix pomatia *(HPA) binds O-linked α-GalNAc but also recognizes Gal (β1–3) GalNAc and α-GlcNAc [9,10]. K_A _values for interactions of HPA with groups containing terminal GalNAc range from 0.1 mM to 5 mM, within the range typical of binding of sugars to lectins [11]. HPA binds to the single O-linked α-GalNAc on the serum macrophage activating factor (GcMAF) [12]. A M13 bacteriophage library consisting of randomized 12-mer sequences at the N-terminus of the pIII protein was screened for an amino acid sequence that would bind to this lectin. Bound phage particles were eluted from the lectin with free GalNAc to ensure specificity of selection. Table 1 lists amino acid sequences of the variable region of phage particles enriched by four cycles of selection, from which a consensus sequence, VQATQSNQHTPR, emerged. [An extensive screen of linear and disulfide-constrained 7-mer phage libraries failed to provide a consensus sequence (data not shown)].

**Table 1:** Amino acid sequences of the N-terminus of protein PIII from separate phage plaques after 4 cycles of selection.

A	**Q**	**A**	L	G	L	S	A	I	S	**P**	**R**
**V**	**Q**	**A**	**T**	**Q**	**S**	**N**	**Q**	**H**	**T**	**P**	**R**
E	**Q**	**A**	**T**	P	R	**N**	H	N	S	**P**	P
**V**	**Q**	**A**	**T**	P	R	L	**Q**	**H**	**T**	**P**	**R**
A	**Q**	G	P	P	**S**	K	**Q**	**H**	S	**P**	P
**V**	**Q**	**A**	I	**Q**	**S**	**N**	**Q**	L	**T**	**P**	**R**
**V**	**Q**	**A**	**T**	T	V	Q	I	Q	H	A	P
**V**	**Q**	**A**	G	**Q**	**S**	**N**	A	**H**	**T**	A	G
**V**	**Q**	**A**	**T**	**Q**	**S**	**N**	**Q**	**H**	**T**	**P**	**R**
**V**	**Q**	**A**	R	**Q**	**S**	**N**	**Q**	**H**	**T**	**P**	**R**
**V**	**Q**	N	Y	**Q**	**S**	**N**	**Q**	**H**	**T**	**P**	**R**
T	F	**A**	**T**	**Q**	**S**	**N**	**Q**	**H**	**T**	**P**	**R**
											
Consensus											
**V**	**Q**	**A**	**T**	**Q**	**S**	**N**	**Q**	**H**	**T**	**P**	**R**

**Figure 1:**
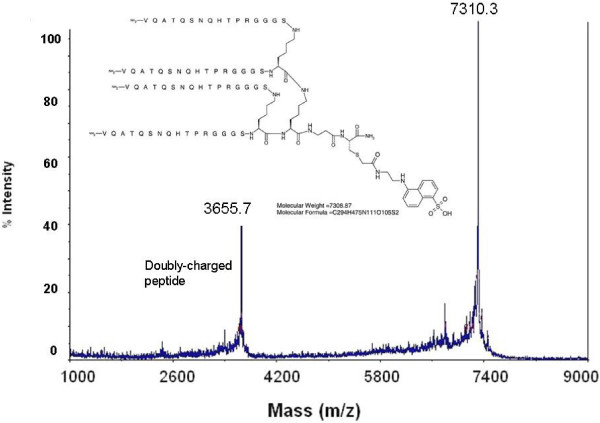
**Structure and mass spectrum of L4**.

The peptide was added to a tri-Lys core [13], which provided four identical sequences within the same structure (designated L4). A GGGS sequence, adjacent to the variable region in the pIII protein, was retained as a spacer to move the mimetic sequence away from the core. A dansyl group was attached to the sulfhydryl group of C-terminal Cys to provide a chromophore for determination of concentration and detection by fluorescence. The structure of the final product was [(VQATQSNQHTPRGGGS)_2_K]_2_K-βA-(S-dansyl)C (Fig. 1). The peptide was also synthesized with the C-terminal βA-(S-dansyl)C replaced with ε-biotinyl-K. All peptides terminated with an amide group at the C-terminus.

The mass spectrum of dansylated L4 contained a signal for the singly-charged (m+1) molecule of 7,310 Da, which was identical to the calculated mass of the protonated quadravalent structure (Fig. 1). The signal at m/z = 3,656 Da corresponded to the doubly-charged peptide.

## Lectin binding

**Figure 2:**
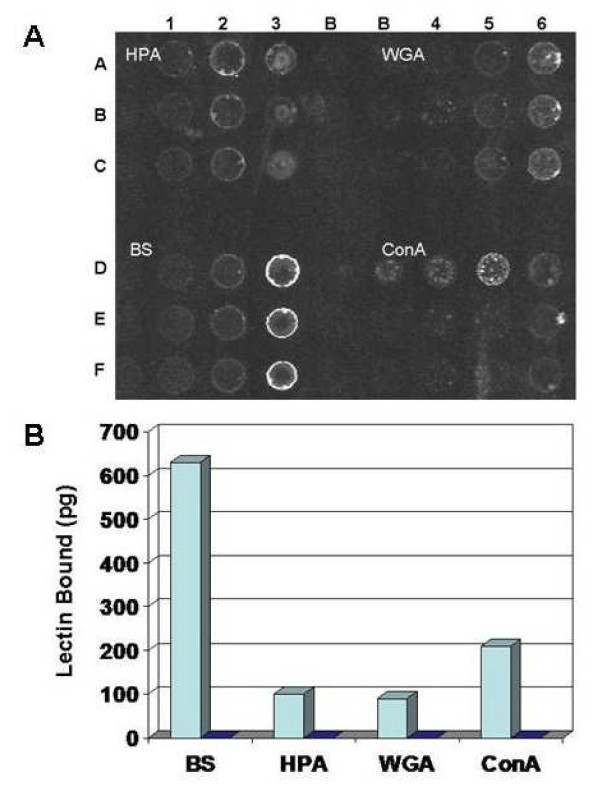
**Binding of lectins to L4**. (**A**) For each lectin, 4, 20 and 100 ng were spotted in triplicate. One set of a total of four sets is shown in the figure. Spots in rows A-C, columns 1–3 contained *H. pomatia *lectin (HPA), rows A-C, columns 4–6 contained *T. vulgare *lectin (WGA), rows D-F, columns 1–3 contained *B. simplicifolia *B_4 _(BS) and rows D-F, columns 4–6 contained *C. ensiformis *lectin (ConA). The slide was incubated with 2% gelatin to block non-specific sites. Biotin-tagged peptide was incubated with the slide and then the amount of peptide bound was detected by binding of Cy3-conjugated streptavidin and measuring fluorescence of the spots. In columns marked B, buffer A was spotted as blanks. (**B**) Biotin-tagged L4 or control peptide was bound in wells of a streptavidin-coated microtiter plate. The wells were blocked with 2% gelatin, washed, and then equal amounts (50 ng) of lectins conjugated with peroxidase were added. After washing, the amount of lectin bound was detected by peroxidase activity. Standard curves were determined with each lectin-peroxidase conjugate to quantitate binding. Data are expressed as the amount of lectin bound minus a blank without peptide. Light blue bars, peptide L4; dark blue bars, control peptide.

Whether L4 expressed characteristics of a sugar was tested by two methods. Lectins whose primary specificities are GalNAc (*Helix pomatia*, HPA); GlcNAc or NeuNAc (wheat germ agglutinin, WGA); Gal [*Griffonia (Bandeiraea) simplicifolia *isolectin B_4_, BS]; and Man or Glc (concanavalin A, ConA), were fixed onto a glass slide. Non-specific sites were blocked with gelatin, and the slide was then incubated with biotin-tagged L4. Binding was detected by adding streptavidin labeled with the fluorescent dye Cy3. Lectins were spotted in triplicate to minimize conclusions from inaccurate delivery, as indicated by the top row of ConA. All four lectins bound L4, with strongest binding found with the Gal-specific lectin, BS (Fig. 2A). Binding was also observed with HPA and with WGA, which binds to clusters of GalNAc [14,15].

In a second assay, biotin-tagged L4 was first bound in wells of a microtiter plate coated with streptavidin, and the extent of binding was measured by activity of horseradish peroxidase conjugated to lectins that were retained in the wells. The Gal-specific lectin BS again bound most extensively to L4 (Fig. 2B). Binding of the lectins to a control peptide, synthesized with the spacer sequence repeated (*i.e*., VGGGSGGGS-), was not detected (Fig. 2B). These binding assays were designed to maintain maximal flexibility of the peptide. When adsorbed onto clean polystyrene surfaces of wells of a microtiter plate, as done in conventional solid-phase binding assays [13,16], the arms of the peptide are most likely immobilized. In such assays, binding of BS was negligible while binding to WGA and ConA increased (data not shown).

## Biological activity of the mimetic peptide

### Cytotoxicity

L4, from 3 nM to 90 μM, did not affect the proliferation of human peripheral blood mononuclear cells (PBMCs) when measured by the incorporation of bromo-deoxyuridine into DNA over a 48-h period (data not shown).

### Cytokine release

Cytokines, which include interleukins (IL's) and chemokines, are regulatory proteins that are released from activated cells and act as intercellular mediators in the generation of a response. A preliminary survey to determine whether L4 induces a change in secretion of cytokines from human PBMCs indicated that IL-15, Eotaxin and TIMP-2 increased approximately two-fold over control levels in the culture medium in response to a 4-h treatment with 100 nM L4. In contrast, the amounts of IL-2 and IL-8 in the medium were approximately half of that in the control culture that received phosphate-buffered saline (PBS), the vehicle, in place of the peptide solution. No significant changes were detected from control samples in the amounts of other cytokines, including IL-1β, IL-6, IL-10, IFN-γ, or TNF-α. After 24 h of treatment, IL-8, G-CSF, I-309 and Eotaxin-2 were significantly elevated in the medium (data not shown).

**Figure 3:**
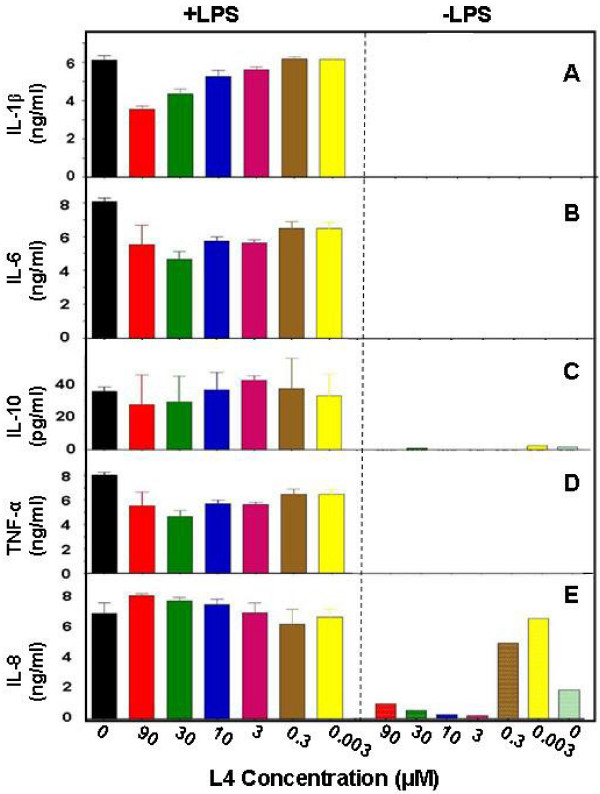
**Quantitative analysis of cytokines**. The amounts of several cytokines in media of PBMC cultures treated for 24 h with L4 in the presence of 1 ng/ml LPS (+LPS) or in the absence (-LPS) were determined. (**A**) IL-1β, (**B**) IL-6, (**C**) IL-10, (**D**) TNF-α, and (**E**) IL-8. The concentration of L4 in each assay is indicated. Assays were done in quadruplicate.

To quantitatively assess effects of L4 on cells, secretion of several key cytokines by PBMCs was assayed after incubation for 24 h with L4 or with lipopolysaccharide (LPS), the prototypic inflammatory agent, as a positive control. LPS (1 ng/ml) strongly stimulated secretion of cytokines IL-1β, IL-6, IL-8, IL-10 and TNF-α. The peptide at relatively high concentrations (*e.g*., 30 μM and 90 μM, Fig. 3A, B, D) weakly but significantly inhibited release of IL-1β, IL-6 and TNF-α induced by LPS. The amounts of these cytokines in media of cultures treated with peptide alone were not significantly different from controls (Fig. 3A to 3D). In contrast, secretion of IL-8 was stimulated by the peptide nearly as strongly as with LPS, with maximal secretion at 3 and 300 nM L4 (Fig. 3E). Higher concentrations, *e.g*., 3 to 30 μM, inhibited release of IL-8 as compared with the untreated, control level (light green bar). IL-8 is a chemokine released from activated macrophages that attracts neutrophils and plays an important role in host defense by enhancing microbicidal activity and cytotoxicity [17,18]. For example, IL-8 dramatically reduced growth of human ovarian cancer cells and mediated effects of the chemotherapeutic drug paclitaxel by targeting neutrophils and monocytes to the tumor [18].

**Figure 4:**
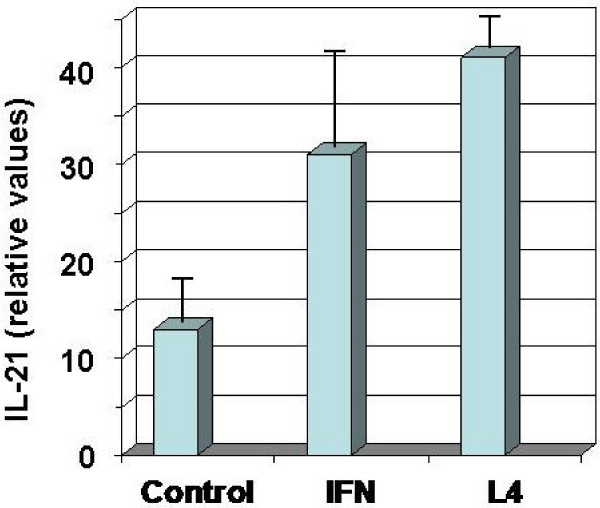
**Stimulation of IL-21 release from PBMCs by treatment with L4**. PBMCs were treated with 1 nM (400 U/ml) IFN-γ or 50 nM L4 for 16 h. The media were blotted onto membranes and stained with chicken anti-IL-21 antibodies and rabbit anti-chicken antibodies conjugated with peroxidase. The graph shows mean ± SEM values of four measurements.

IL-21, a cytokine secreted from activated T cells, is of particular interest in regard to its pleiotropic immunomodulatory and anti-tumor activities [19,20]. A 16-h treatment of PBMCs with 50 nM L4 induced as strong a release of IL-21 as a highly effective concentration [1 nM (400 U/ml)] of IFN-γ, a known stimulant of IL-21 release [19] (Fig. 4). The low control value indicated that the peptide expressed an activity not provided by proteins and other factors from serum in the culture medium. IL-21 is most homologous to IL-15 and to a lesser extent IL-2. IL-21 has activity on all leukocyte subsets, including dendritic cells and monocytes [20]. IL-15 activates T cells and promotes long-term survival of memory T cells [21].

The control peptide, with the mimetic sequence replaced with VGGGSGGGS-, did not induce release of IL-8, IL-21 or other cytokines including IL-1β, IL-6, IL-10, IFN-γ and TNF-α, (data not shown).

## Conclusion

The multivalent peptide induced a remarkable pattern of cytokine release. Secretions of several cytokines that are used singly in clinical practice were stimulated, while release of highly inflammatory cytokines, such as IL-1β, IL-6 and TNF-α, did not appear to be changed.

## Methods

### Phage display library screening and DNA sequencing

The Ph.D.™-12 phage display library (New England BioLabs, Ipswich, MA) was mixed with HPA conjugated to agarose beads (Sigma-Aldrich, St. Louis, MO) at room temperature in 200 μl of 50 mM Tris-HCl (pH 7.5) containing 150 mM NaCl, 1 mM CaCl_2_, 1 mM MgCl_2 _and 1 mM MnCl_2 _(buffer A). The beads were washed three times and suspended in 1 ml of buffer A. GalNAc was added to the suspension to 100 mM and after 10 min the beads were removed by centrifugation. Phage released from the lectin were amplified by infection of *E. coli *strain ER2738 (New England BioLabs) and the screen was repeated three times. The buffer for the last cycle included 0.5% (w/v) Tween 20. Phage released by GalNAc were plated on a lawn of *E. coli *and phage in individual plaques were amplified and DNA was sequenced with an Applied Biosystems 3730 Analyzer (Applied Biosystems, Foster City, CA) with the -96 gIII primer.

### Synthesis of peptides

Quadravalent peptides were synthesized on a tri-Lys core [13] utilizing Fmoc (9-fluorenylmethoxycarbonyl)-protected amino acids and a Milligen Biosearch 9050+ continuous flow peptide synthesizer (Millipore, Billerica, MA). The C-terminus consisted of βAla(βA)-Cys(C). A dansyl group was incorporated by reaction of the thiol group on C-terminal Cys with 5-((((2-iodoacetyl)amino)ethyl)amino)naphthalene-1-sulfonic acid (IAEDANS) (Molecular Probes, Eugene, OR). Biotin was incorporated into the peptide with C-terminal ε-biotinyl-Lys in place of βAla-Cys. A control peptide was synthesized with the sequence identified by phage display replaced with a repeat of the spacer sequence (*i.e*., VGGGSGGGS-). Peptides were purified on a preparative Jupiter Proteo C12 column (21.2 mm × 250 mm) (Phenomenex, Torrance, CA) using a gradient from 8% to 18% acetonitrile in water containing 0.1% TFA, dried under vacuum, dissolved in sterile PBS, pH 7.2, and passed through a Sephadex G-25 or G-15 column (1 × 45 cm) in PBS to remove TFA. Concentration was determined by absorbance of the dansyl group (extinction coefficient, ε_mM _= 5.7 cm^-1 ^at 336 nm) or by the bicinchoninic acid assay (Pierce, Rockland, IL) using known concentrations of the dansylated peptide as standard. Mass spectroscopy was performed with a Voyager DE STR mass spectrometer (Applied Biosystems).

### Lectin binding

Horseradish peroxidase-conjugated lectins (Sigma-Aldrich) were prepared at a concentration of 500 μg/ml in buffer A and 1 μl of each was spotted 4 times in triplicate onto a glass slide (Full Moon BioSystems, Sunnyvale, CA) at dilutions of 1:5, 1:25 and 1:125. Buffer A was spotted as blanks. The slide was blocked with 2% gelatin in buffer A, and biotin-tagged L4 in buffer A was incubated with the slide overnight at room temperature. The slide was washed 3 times with buffer A and then incubated with streptavidin conjugated with Cy3 (Sigma-Aldrich) for 1 h. The slide was again washed 3 times with buffer A and fluorescence detected in a microarray reader.

For microtiter plate assays, 100 μl of 2 μM biotin-tagged L4 in PBS was added to each well of a high-binding-capacity streptavidin-coated plate (Pierce) and incubated overnight at 4°C. The wells were blocked with 2% gelatin in PBS for 3 h at room temperature, washed 1 time with PBS and then with buffer A. Lectins were added at a concentration of 1 μg/ml in buffer A. After 1 h incubation, wells were washed 4 times with buffer A containing 0.01% Tween-20, 50 μl peroxidase substrate (1-Step Ultra TMB-ELISA, Pierce) was then added, and after 5 min the reaction was stopped with 50 μl 2 M H_2_SO_4_. Absorbance was read immediately at 450 nm. Standard curves were obtained by assaying known amounts of the horseradish peroxidase-conjugated lectins (OD_450_/ng protein).

### Cytokine assays

Human PBMCs (Cellular Technology Ltd, Shaker Heights, OH) were plated at 4 × 10^5 ^cells per well in RPMI 1640 medium containing 10% fetal bovine serum (FBS). After 2 days at 37°C in 5% CO_2_, the medium was replaced and peptide or LPS added and the incubation continued for 24 h. Media were then withdrawn and cytokines assayed with BioSource kits (Invitrogen, Carlsbad, CA). For IL-21 analyses, media were applied to Immobilon-P membranes (Millipore) in a slot-blot apparatus, the membrane was blocked with 5% nonfat dried milk in PBS, incubated with a primary chicken polyclonal anti-IL-21 antibody (Abcam, Cambridge, MA) and then with a rabbit polyclonal anti-chicken IgY antibody (Abcam) conjugated with horseradish peroxidase. Bound antibodies were detected with 3,3',5,5'-tetramethylbenzidine substrate (Sigma-Aldrich). Membranes were scanned with a densitometer to quantitate the extent of reaction.

## Abbreviations

Gal: galactose; GalNAc: N-acetylgalactosamine; G-CSF: granulocyte colony-stimulating factor; Glc: glucose; GlcNAc: N-acetylglucosamine; IFN: interferon; Man: mannose; NeuNAc: sialic acid; PBMCs: peripheral blood mononuclear cells; PBS: phosphate-buffered saline; TNF: tumor necrosis factor.

## Authors' contributions

LLE conceived of the concept of the peptide mimetic, designed the peptides, and performed most of the experimental work. LLE and JKH conducted biochemical studies and drafted the manuscript. The authors declare that they are inventors of technology owned by Arizona State University and the Arizona Board of Regents.
